# A Case of Inguinal Aneurism Treated Successfully by a Ligature to the External Iliac Artery

**Published:** 1842-04-09

**Authors:** Edward Peace

**Affiliations:** One of the Surgeons to the Pennsylvania Hospital


### MEDICAL EXAMINER.

NEW SERIES.

No. 15.] PHILADELPHIA, APRIL 9, 1842. [Vol. I.

*A Case of Inguinal Aneurism treated successfully by a ligature to the External Iliac Artery.* By Edward Peace, M. D., one of the Surgeons to the Pennsylvania Hospital.

John Erwin, a seaman, aged 28 years, of robust constitution and of healthy parents, was admitted into the Pennsylvania Hospital July 17th, 1841, with inguinal aneurism on the right side. Four months previous to this period, after a fall received during a severe and long contested wrestling-match he had severe pain in right groin, which however left him in the course of a few days. He then continued well for two months, suffering only from occasional uneasiness at the knee, at the end of which time there was a return of the pain in his groin—he then observed for the first time a small tumour of the size of a walnut, which has continued to increase in size and accompanied with such an increase of pain as to have incapacitated him from walking during the last three days. The patient thinks that the tumour has increased one-half in size during the last seven days. He called upon an apothecary for advice, who applied forty leeches and recommended hot poultices.

Upon examining the patient on the afternoon of his entrance into the Hospital, we found a pulsating tumour occupying the course of the right femoral artery, extending from one inch above Poupart's ligament to three and a half inches below it. The transverse diameter of the tumour was four inches.

Compressing the aorta arrested the pulsation of the aneurism, but without producing any diminution of its volume.

The skin over the tumour is reddened; pits; and the pain is deep-seated and very severe. The inner side of the knee is also the seat of great pain; the whole limb is swollen; the sensibility is natural except upon the anterior surface of the thigh. No sleep for two nights. Ordered the limb to be slightly elevated, and lead water with laudanum applied to the tumour. A tea-spoonful of solution of morphia procured him a more comfortable night than he had passed for some time.

July 23.—Six days have now elapsed since the patient's entrance into the hospital. All signs of local inflammation have left him; the skin has resumed its natural appearance; the pulsation is not so strong in the tumour, which has increased rapidly in size.

After a consultation with my friend and colleague Dr. Norris, it was determined to apply a ligature to the external iliac artery on the following day. Ordered Ol. Ricini ℥i; also, seventy drops of laudanum to be given two hours before the operation.

July 24th.—Before the medical class, a curved incision, four inches in length, with its convexity directed towards Poupart's ligament, was made through the skin, commencing at a point one inch and a half above and one inch on the inner side of the anterior superior process of the ilium, and terminating half an inch above the situation of the external ring. The arteria ad cutem was divided and tied. The tendon of the external oblique was then divided upon a director. This brought into view the lower edge of the internal oblique and transversalis muscles, which were separated from Poupart's ligament with the handle of the scalpel. It was found necessary to divide some of the fleshy fibres of the transversalis, in order to allow more room for manipulation, as the tumour extended further into the iliac fossa than we supposed. The peritoneum was easily raised up, and the artery was felt beating distinctly, but faintly, contrasting forcibly with the violent vibration of the tumour. The artery, which appeared healthy, was separated from the vein by the finger nail, and a round silk ligature was applied with great facility, by means of the excellent aneurismal needle invented by Professor Gibson of this city. The ligature was applied as high as possible, so as to allow sufficient space for the formation of a coagulum above the epigastric artery, and both ends of the ligature were allowed to remain hanging from the wound, to serve as a drain. The pulsation of the tumour was immediately arrested. The lips of the wound were brought together by two strips of adhesive plaster, and dressed with lint spread with cerate. The patient made no complaint during the operation, which occupied eighteen minutes, but at its termination, his countenance exhibited great distress and anxiety. He was carried to his bed;—his limb slightly elevated. The pulse before the operation was eighty; immediately afterwards seventy-six; the thermometer ninety-four, being the hottest day of the season.

1 o'clock.—Toes of right foot cold and moist; the rest of the limb warm. Two hours afterwards, the coldness extended up to the instep; temperature of both limbs the same; pulse sixty-six.

6 o'clock, P. M.—Pain of the whole limb; florid; pulse seventy-four; skin moist; thirst; no extension of coldness above instep, where the sensibility is very obtuse. Ordered sol. morph. ʒij; iced barley water. Foot enveloped in carded wool.

Sunday, 11 A. M.—Slept well for two hours and awoke in a fright; pain of groin intensely severe; hot fomentations, and sol. morph. ʒij gave much relief—this morning the pain is moderate; pulse seventy-six; temperature of room eighty-eight—between toes of right foot eighty-six—right thigh ninety-six—left thigh ninety-three. Ordered oat-meal gruel and toast-water.

10 P. M.—Pain occasionally darts through the tumour—same relative difference of heat as in the morning. Ordered sol. morph. ʒi.

26th, 11 A. M.—Slept well the whole night. Pulse seventy; skin moist; expression natural; difference of temperature still in favour of right limb; sensibility of the limb improving, but less at the anterior and inner side of the thigh. The warmth of the foot has returned as low as the toes.

27th.—Slept well; both limbs of same temperature; sensibility natural everywhere, with the exception of last phalanx of right toes, which remains cold. A small red spot at the inner side of patella is very painful, prominent and soft; the tumour is also painful and inflamed. Ordered evaporating lotion; sol. morph. ʒi.

28th.—No sleep; urination frequent during yesterday, and every fifteen minutes during the night; knee less painful, also the tumour, which is now evidently smaller; the skin wrinkling and pale. Ordered flaxseed tea and a large cataplasm over pubic region.

29th.—Passed a comfortable night; has urinated but twice since yesterday; feels perfectly well, with the exception of some uneasiness of right limb. Examined the wound, which is suppurating freely. Two-thirds of the incision have united by the first intention. Left the wound open, as it is closing too rapidly, and made use of a simple dressing. The ligature of the superficial artery came away.

No dejection since the operation. Ordered a common enema, which produced a copious and healthy evacuation.

August 3d.—Wound healthy; the tumour diminishes in size and is firmer; the œdema of limb entirely gone.

The patient continued to improve without any unpleasant symptoms; the aneurism becoming smaller and firmer till August the 24th, thirty days after the operation, when the ligature came away, having a large loop. The wound is now closed with the exception of the point whence the ligature issued.

September 15th.—The wound not yet closed; probed it, and found a sinus extending an inch and a half in depth. Enlarged the sinus, filled it with lint, and applied a poultice.

22d.—Wound cicatrized, tumour lessening daily and quite firm.

30th.—Tumour half of its former size. The patient walks about his room, and can bear his whole weight upon the affected side.

November 24th.—Discharged from the hospital; is able to return to his work. The tumour is now about the size of a walnut.

A month afterwards came to see me, preparatory to his going to sea as mate of a vessel, perfectly restored.

*Remarks.*—Owing to the rapid progress of the aneurism, it was not deemed proper to apply pressure above the tumour with a view of dilating the collateral branches, as recommended by several eminent surgeons; and it may be well questioned whether the benefit resulting from dilatation of the vessels which are to nourish the limb will ever compensate for the greater danger incurred by an increase of the tumour and inflammation of the surrounding tissues. The same reasons may be urged against the recommendation to promote a cure by pressure over the tumour; as was practically exemplified in a case of inguinal aneurism reported by Dr. Post in the Amer. Med. and Physical Register, (New York) vol. iv., where the patient, apprehending more from an operation than from the disease, refused the application of a ligature.

His surgeon then directed the application of a compress and bandage to keep up a constant and moderate pressure upon the tumour. Under this treatment the aneurism diminished for a time, but then increased rapidly, while severe pain and considerable local inflammation and tumefaction of the upper part of the thigh supervened. These symptoms were finally relieved by a removal of their cause, and a resort to cold applications and evacuants. When the patient at last submitted to an operation, it was found impracticable to separate the peritoneum,—which is ordinarily so easy,—owing to the adhesions that had taken place between that membrane and Poupart's ligament from the previous inflammation. The surgeon was obliged to cut through the peritoneum in order to apply the ligature; consequently, the patient was exposed to the additional hazard of inflammation of that membrane. Fortunately, the termination of the case was favourable.

The next point of consideration was the propriety of applying one ligature or two. Owing to repeated failures when but one ligature is applied, some modern surgeons have recommended a return to the old method of securing the artery above and below the tumour. Whether the greater certainty of preventing a return of the circulation by these means makes up for the increased danger of a double operation, can only be tested by statistical information which is not easily obtained, this difficulty resulting from the small number of cases that have occurred in the practice of any one surgeon or at any one place and from the suppression of unsuccessful cases. With a view of contributing to the amount of knowledge bearing on this question, the experience of this city is here given. The ligature of the external iliac, for aneurism of the femoral artery, has been successfully performed by Dr. Dorsey in 1811; by Dr. Randolph in 1825 ; and in the third case by Professor Horner at the Blockley Hospital, where two ligatures were applied and the sac opened. The particulars of this case, the termination of which was unfavourable, will be soon reported. The shortness of this catalogue shows the extreme rarity of the disease. Recollecting that even a return of the pulsation does not necessarily prevent the formation of coagula and a final cure, it appears to be most prudent to trust to one ligature in aneurisms of the first class, whilst in smaller vessels, where there is but little danger of a fatal termination, two ligatures will more certainly prevent a return of the circulation.

It has not occurred in any prior case that I have noticed, that the temperature of the limb operated upon has been greater than that of the sound limb, immediately after the operation. This difference of heat in favour of the unsound limb continued for three days, nor was it at any time reversed. Dr. Neil, who was residing at that time in the hospital, kept an exact observation of these facts, as well as myself. They exemplified in a remarkable degree the importance of the capillary circulation, and the power of the collateral branches, to compensate for obliteration of the main artery ; and they would lead us to suspect that, in this case, the impetus of the circulation had been diminished by a partial formation of coagula. This would explain why we were unable to reduce the size of the tumour when we arrested the pulsation by pressure upon the aorta, and the singular feebleness of the pulsation in the artery which was remarked when the ligature was applied.

With the exception of the irritation of the bladder on the fifth day, no unpleasant symptoms of any kind occurred during the whole course of treatment, which was unnecessarily prolonged by the ligature not having been tied sufficiently tight. This was manifested by the large size of the loop, and it satisfactorily accounts for the retention of the ligature for thirty days.

